# Time-Intensity Curve parametric imaging as a novel quantitative biomarker: enhancing diagnostic accuracy and inter-rater reliability in prostate cancer ultrasound

**DOI:** 10.3389/fonc.2025.1644411

**Published:** 2025-10-09

**Authors:** You Zhou, Mingyou Liu, Jiguang Zhou, Hanzong Lin, Jinxin Lan, Yusheng Xu, Xiao Yang, Ming Chen

**Affiliations:** ^1^ Department of Ultrasound, Zhangzhou Affiliated Hospital of Fujian Medical University (Zhangzhou Municipal Hospital of Fujian Province), Zhangzhou, China; ^2^ Information Center, Peking Union Medical College Hospital, Beijing, China; ^3^ Department of Ultrasound Chinese Academy of Medical Sciences and Peking Union Medical College, Beijing, China

**Keywords:** prostate cancer, contrast-enhanced ultrasound (CEUS), Time-Intensity Curve (TIC), parametric imaging, quantitative ultrasound, diagnostic accuracy, angiogenesis

## Abstract

**Objective:**

To investigate the diagnostic utility of a novel Time-Intensity Curve (TIC) parametric imaging technique for improving the accuracy of prostate cancer (PCa) detection. This study aimed to quantitatively assess the technology’s impact on the diagnostic performance of ultrasound physicians with disparate levels of clinical experience and to evaluate its potential to standardize diagnostic interpretation.

**Methods:**

We conducted a retrospective analysis of 62 patients who underwent transrectal contrast-enhanced ultrasound (TR-CEUS) at Zhangzhou Affiliated Hospital of Fujian Medical University between December 2024 and March 2025. All diagnoses were confirmed by systematic 12-core prostate biopsy. A proprietary TIC parametric imaging software was used to perform a pixel-wise analysis of CEUS cineloops, generating quantitative maps of the perfusion parameter “mean gradient to peak.” These maps were then qualitatively classified based on the spatial heterogeneity of perfusion into a four-tier discreteness system. Four junior physicians (1–2 years experience) and four senior physicians (>10 years experience) independently evaluated patient cases, first using conventional grayscale and CEUS images, and then again after a washout period with the addition of the TIC parametric maps. A paired chi-square test compared diagnostic outcomes. Inter-rater and intra-rater reliability were assessed using intra-class correlation coefficients (ICC). Diagnostic performance was evaluated using Receiver Operating Characteristic (ROC) curve analysis, with Area Under the Curve (AUC) as the primary metric.

**Results:**

A paired chi-square test demonstrated a statistically significant improvement in diagnostic accuracy when TIC parametric imaging was used as an adjunct to conventional ultrasound (p < 0.0001). The introduction of TIC maps markedly improved intra-group diagnostic consistency; the ICC for junior physicians increased from a good 0.832 to an excellent 0.915, and for senior physicians, it rose from an excellent 0.878 to a near-perfect 0.941. Most notably, the diagnostic performance gap between experience levels was effectively eliminated. The AUC for junior physicians surged from 0.43 (95% CI: 0.36-0.50) to 0.85 (95% CI: 0.79-0.90; p < 0.0001). For senior physicians, the AUC improved from 0.53 (95% CI: 0.46-0.60) to an outstanding 0.95 (95% CI: 0.92-0.99; p < 0.0001). With TIC assistance, the diagnostic efficacy of both junior and senior physicians converged at a high level of performance.

**Conclusion:**

TIC parametric imaging, through its ability to objectively quantify and visualize the spatial heterogeneity of tumor blood perfusion, serves as a powerful adjunctive tool that significantly enhances the accuracy and consistency of prostate cancer diagnosis. This technology demonstrates profound clinical value by substantially mitigating the influence of operator experience, thereby shortening the learning curve for novice physicians and standardizing diagnostic quality across all levels of expertise. the sample size is relatively small, which can lead to wide sensitivity confidence intervals and increases the risk of statistical anomalies. require validation in larger, multi-center prospective trials.

## 1 Introduction

### 1.1 The global burden and diagnostic challenges of prostate cancer

Prostate cancer (PCa) represents a formidable global health challenge. It stands as the second most frequently diagnosed cancer and the fifth leading cause of cancer mortality among men worldwide (1). Its incidence exhibits significant geographic variation, largely influenced by the prevalence of Prostate-Specific Antigen (PSA) screening and differences in lifestyle, genetics, and environmental factors. In developed nations, particularly in North America and Europe, PCa is the most common non-cutaneous cancer in men, driven by widespread screening programs that detect a high number of early-stage, localized tumors. However, this has led to a persistent and contentious debate surrounding overdiagnosis—the detection of indolent cancers that would never have become clinically significant—and the consequent risks of overtreatment, which include erectile dysfunction, urinary incontinence, and bowel complications (2, 3).

The cornerstone of PCa detection for decades has been a combination of digital rectal examination (DRE) and serum PSA testing. While the PSA test has undoubtedly increased the detection of early-stage disease, its utility is severely hampered by low specificity. Elevated PSA levels are not exclusive to cancer and are frequently observed in benign conditions such as benign prostatic hyperplasia (BPH) and prostatitis, leading to a high rate of false positives and subsequent unnecessary prostate biopsies (4). This diagnostic ambiguity has fueled an intensive search for more specific and reliable biomarkers and imaging modalities to improve risk stratification and guide clinical decision-making.

### 1.2 The evolving landscape of prostate imaging

Conventional grayscale transrectal ultrasound (TRUS) has long been the workhorse for guiding prostate biopsies but possesses poor intrinsic accuracy for cancer detection. Most prostate cancers are isoechoic to the surrounding benign tissue, rendering them invisible on standard B-mode imaging (5). This limitation led to the development of the systematic 12-core biopsy protocol, a spatially distributed but essentially blind sampling method with a significant risk of sampling error, potentially missing clinically significant tumors or misclassifying tumor grade.

To overcome these limitations, a suite of advanced ultrasound techniques has been introduced. Doppler ultrasound can identify areas of increased blood flow, a hallmark of malignant tumors, but it is often confounded by the hypervascularity associated with inflammation. Contrast-enhanced ultrasound (TR-CEUS) utilizes microbubble contrast agents to visualize tissue microvasculature in real-time. It has shown promise in identifying hypervascular lesions suggestive of PCa, yet its interpretation remains subjective and can be compromised by the diffuse hyperperfusion seen in BPH and prostatitis, leading to persistent challenges with false positives (6). Ultrasound elastography assesses tissue stiffness, capitalizing on the principle that cancerous tissue is typically firmer than benign tissue. However, its efficacy is limited by a lack of operator reproducibility and confounding factors such as prostatic calcifications, fibrosis, and the pressure applied by the operator (7).

More advanced techniques like three-dimensional TRUS (3D-TRUS) and micro-ultrasound (Micro-US) have also emerged. 3D-TRUS allows for a more comprehensive anatomical assessment but requires complex image registration and expensive equipment, hindering its widespread adoption (8). Micro-US, operating at a much higher frequency (~29 MHz), offers superior spatial resolution for visualizing prostatic microarchitecture. While promising for identifying suspicious lesions, it is highly operator-dependent and its utility in scanning the entire gland, particularly the anterior prostate, remains a challenge (9).

In parallel, multi-parametric Magnetic Resonance Imaging (mpMRI) has revolutionized the PCa diagnostic pathway. By combining T2-weighted imaging with functional sequences like Diffusion-Weighted Imaging (DWI) and Dynamic Contrast-Enhanced (DCE) MRI, mpMRI provides a comprehensive morphological and functional assessment. The Prostate Imaging-Reporting and Data System (PI-RADS) standardizes interpretation and has proven effective in identifying clinically significant PCa, guiding targeted biopsies, and monitoring patients on active surveillance (10). However, mpMRI is expensive, not universally accessible, time-consuming, and has contraindications for some patients (e.g., those with certain metallic implants or severe renal impairment).

### 1.3 The rationale for quantitative perfusion imaging

A fundamental biological hallmark of solid tumors, including prostate cancer, is angiogenesis—the formation of new blood vessels from pre-existing ones. This process is essential for tumor growth, invasion, and metastasis (11). Tumor-associated neovasculature is structurally and functionally abnormal, characterized by tortuosity, chaotic organization, and increased permeability. These pathological changes result in altered tissue hemodynamics, which can be interrogated using dynamic imaging techniques.

TR-CEUS provides a window into these hemodynamic changes. Following a bolus injection of microbubbles, their transit through the prostatic microvasculature can be recorded. Subsequent analysis of the time-intensity curve (TIC) within a manually placed region of interest (ROI) can yield quantitative perfusion parameters. This approach, known as quantitative CEUS (qCEUS), can measure parameters like peak intensity (reflecting blood volume), time to peak (reflecting flow velocity), and wash-in rate (12). However, conventional qCEUS is fundamentally limited. Its reliance on manually drawn ROIs introduces significant subjectivity and sampling bias. The analysis is confined to a small, pre-selected area, failing to capture the perfusion characteristics of the entire gland or the spatial heterogeneity that is a key feature of tumor biology. A tumor is not a homogenous mass; it is a complex ecosystem with areas of high proliferation and vascularity interspersed with regions of hypoxia and necrosis. A single ROI cannot capture this complexity.

### 1.4 Study hypothesis and aims

This study introduces and evaluates a novel TIC parametric imaging technique designed to overcome the limitations of conventional CEUS and ROI-based analysis. By performing a TIC analysis on a pixel-by-pixel basis for the entire ultrasound image plane, this method generates a quantitative parametric map that visually encodes the perfusion characteristics across the entire prostate section. This approach transforms a dynamic cineloop into a single, intuitive, color-coded static image, revealing the spatial distribution of blood flow and highlighting areas of abnormal perfusion heterogeneity.

We hypothesized that this objective, comprehensive visualization of perfusion heterogeneity would significantly improve diagnostic accuracy and reduce the variability in interpretation among physicians. Therefore, the primary aims of this study were:

To determine if TIC parametric imaging, as an adjunct to standard TR-CEUS, improves the diagnostic accuracy for prostate cancer compared to standard TR-CEUS alone.

To quantify the impact of this technology on the diagnostic consistency (inter-rater reliability) among physicians within the same experience group (junior and senior).

To assess whether TIC parametric imaging can bridge the diagnostic performance gap between junior and senior physicians, potentially shortening the learning curve for PCa diagnosis.

## 2 Materials and methods

### 2.1 Study design and patient cohort

This study was a retrospective, single-center, diagnostic accuracy study conducted with the approval of the Medical Ethics Committee of Zhangzhou Affiliated Hospital of Fujian Medical University (Zhangzhou Municipal Hospital of Fujian Province) (Approval No. 2025LWB167). The study adhered to the principles of the Declaration of Helsinki. All patients had previously provided written informed consent for the clinical procedures (CEUS and biopsy) and for the use of their anonymized data for research purposes.

We retrospectively reviewed the records of 70 consecutive patients who underwent TR-CEUS followed by prostate biopsy at our tertiary care academic medical center between December 2024 and March 2025. Patients were eligible for inclusion if they met the following criteria: ① underwent TR-CEUS immediately followed by a 12-core systematic TRUS-guided prostate biopsy, ensuring a definitive histopathological reference standard; ② had no prior history of prostate surgery (e.g., transurethral resection of the prostate), radiation therapy, or other manipulations that could alter prostatic anatomy and vascularity; and ③ had a serum total PSA concentration in the range of 4 to 150 ng/ml, a cohort in which diagnostic uncertainty is common.

Exclusion criteria were stringently applied: ① CEUS cineloop images of suboptimal quality (e.g., due to significant motion artifacts, poor probe contact, or incomplete contrast wash-in/wash-out) that would preclude reliable quantitative analysis; ② patients who had not undergone a prostate MRI prior to the CEUS examination (this was a local protocol consideration, though MRI data was not used in the primary analysis); and ③ patients with a known history of allergy or hypersensitivity to the ultrasound contrast agent (SonoVue^®^). After applying these criteria, 8 patients were excluded, resulting in a final study cohort of 62 patients.

### 2.2 Ultrasound image acquisition protocol

All ultrasound examinations were performed by one of two senior sonographers, each with over 10 years of experience in prostate imaging, to ensure consistency in the acquisition technique. A Mindray Resona 9s ultrasound system (Mindray Medical International, Shenzhen, China) equipped with a high-frequency ELC13-4U biplane transrectal probe (frequency range: 4–13 MHz) was used for all procedures.

Patients were placed in the left lateral decubitus position. A baseline grayscale TRUS examination was first performed in both sagittal and axial planes to assess prostate volume, identify calcifications, and note any hypoechoic lesions. The imaging plane for CEUS was standardized to the medial sagittal view of the prostate, which is a key plane used in the standard 12-core biopsy scheme.

For the CEUS portion, the system was switched to a contrast-specific imaging mode. The mechanical index (MI) was maintained at a low level (0.04 to 0.13) to minimize microbubble destruction and ensure accurate perfusion assessment. The ultrasound focus was placed at the base of the prostate. A 2.4 mL bolus of the ultrasound contrast agent SonoVue^®^ (Bracco, Milan, Italy), reconstituted from 59 mg of sulfur hexafluoride microparticles, was administered intravenously via a 20-gauge cannula in an antecubital vein, followed immediately by a 5 mL saline flush. A digital cineloop of the entire contrast transit phase, from initial wash-in to late-phase washout, was recorded for a duration of at least 120 seconds. The acquired grayscale and CEUS cineloop data were then exported and archived in the standard DICOM format for offline analysis.

### 2.3 Histopathological reference standard

Immediately following the CEUS procedure, all 62 patients underwent a TRUS-guided 12-core systematic prostate biopsy performed by the same senior physician. The standard sextant protocol was employed, with biopsies taken from the apex, mid-gland, and base of the prostate on both the right and left sides, targeting the peripheral zone. Each core was meticulously labeled according to its anatomical location and placed in a separate formalin container. The specimens were then processed and analyzed by an experienced genitourinary pathologist who was blinded to the CEUS findings. The final pathology report, indicating the presence or absence of adenocarcinoma and the Gleason score for malignant cases, served as the definitive reference standard for this study.

### 2.4 TIC parametric imaging and feature analysis

The core of our methodology involved offline post-processing of the stored DICOM CEUS cineloops using a specialized software package: Time Intensity Curve Tool (v0.2.4 Beta, Collaborative Enhanced Tomography, Beijing, China).

The analysis workflow was as follows:

Motion Correction: An initial motion compensation algorithm was applied to the cineloop to correct for minor patient movements and ensure that each pixel corresponded to the same anatomical location throughout the dynamic sequence.

ROI Delineation: A senior physician (>10 years experience), blinded to the final pathology, manually delineated the ROI by tracing the entire boundary of the prostate on a representative frame of the sagittal view. This whole-gland approach was chosen to ensure a comprehensive, unbiased assessment, in contrast to traditional small-ROI methods.

Pixel-wise TIC Generation: The software then automatically generated a TIC for every single pixel within the delineated prostate boundary. Each TIC plots the signal intensity (proportional to microbubble concentration) at that pixel against time.

Parametric Calculation: From each pixel’s TIC, the software calculated a variety of quantitative perfusion parameters. For this study, we focused on the mean gradient to peak. This parameter represents the average slope of the TIC during the wash-in phase (from the start of enhancement to the point of peak intensity). It reflects the average rate of microbubble accumulation and is a robust indicator of the velocity and efficiency of tissue perfusion. It was chosen for its ability to combine both temporal and intensity information into a single, descriptive metric of perfusion dynamics.

Parametric Map Generation: The calculated “mean gradient to peak” value for each pixel was then color-coded and displayed as a static, two-dimensional parametric map overlaid on the grayscale image. The color scale ranged from cool colors (e.g., blue), indicating slow perfusion, to hot colors (e.g., red), indicating rapid perfusion.

**Figure 1:**
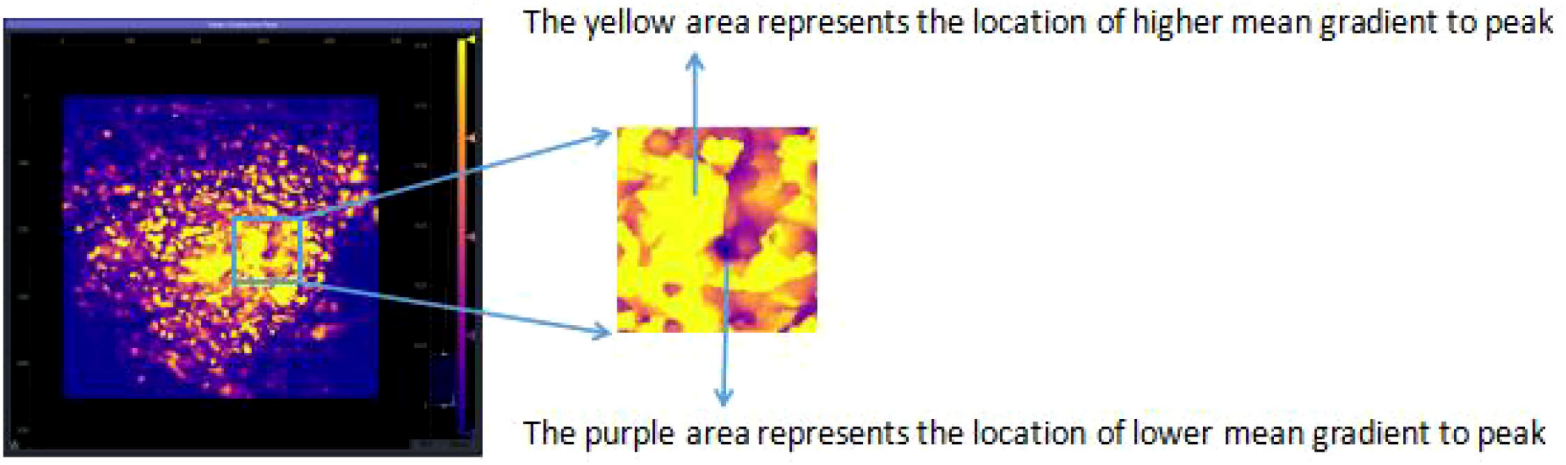
Schematic diagram of average peak enhancement rate parameters.

**Figure 2:**
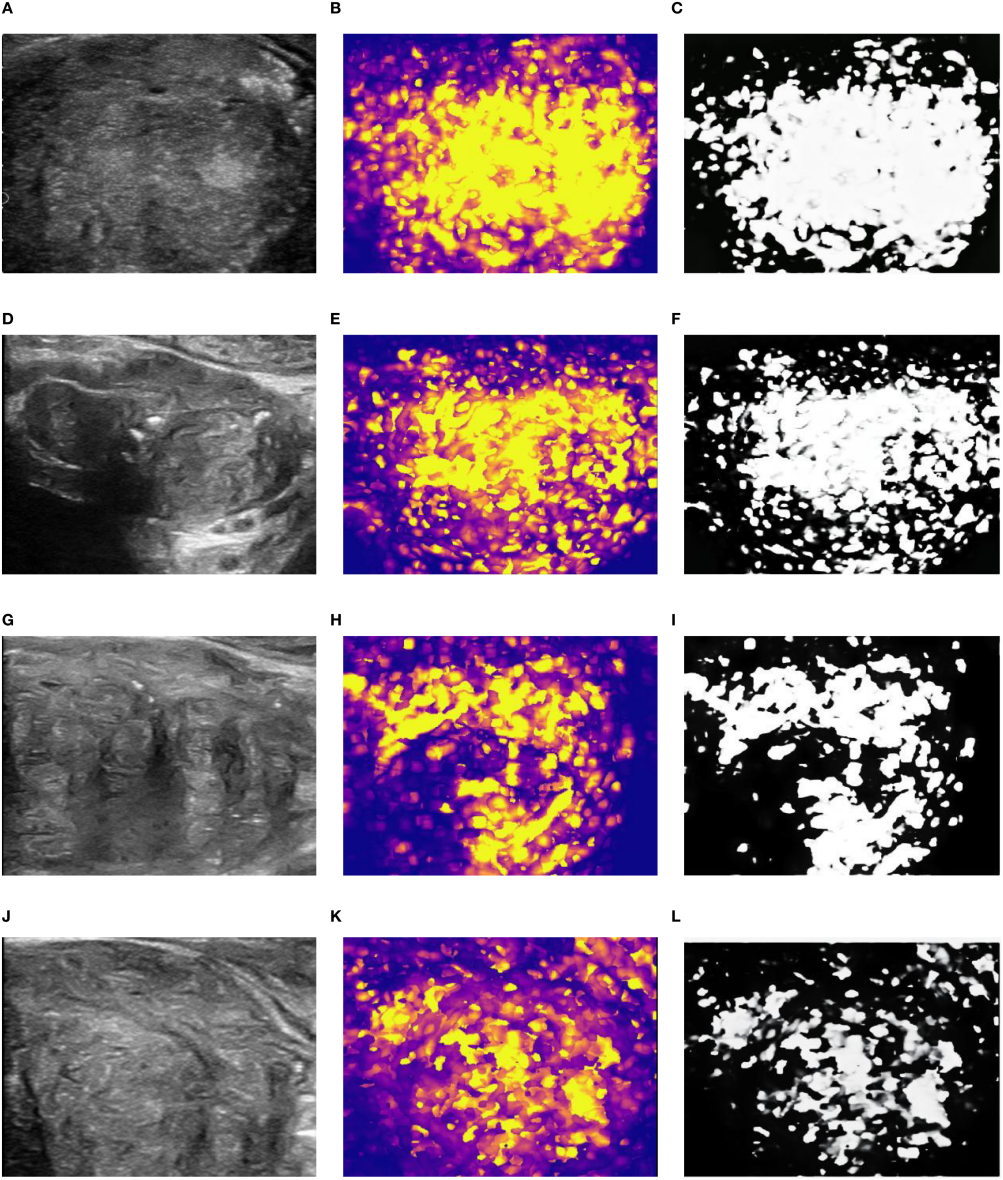
Grading of spatial distribution characteristics of DIC parameters based on average peak enhancement rate. **(A-C)** 2D diagram, parameter diagram and classification diagram of type 1; **(D-F)** 2D diagram, parameter diagram and classification diagram of type 2; **(G-I)** 2D diagram, parameter diagram and classification diagram of type 3; **(J-L)** 2D diagram, parameter diagram and classification diagram of type 4.

Qualitative Heterogeneity Classification: Finally, these quantitative maps were qualitatively assessed for their spatial pattern of perfusion. Based on the distribution, or discreteness, of the hyper-perfused (hot color) regions, each map was classified into one of four types (as illustrated conceptually in the original study’s **Figures 1**, **2**):

Type 1 (Completely Concentrated): A single, focal, contiguous area of high perfusion.

Type 2 (Predominantly Concentrated): One dominant area of high perfusion with a few small, scattered satellite areas.

Type 3 (Predominantly Discrete): Multiple, distinct, non-contiguous areas of high perfusion, with some additional scattered foci.

Type 4 (Completely Discrete): Numerous small foci of high perfusion scattered diffusely and chaotically throughout the prostate gland.

### 2.5 Diagnostic reading sessions and experimental design

A paired, crossover reader study was designed to rigorously evaluate the impact of the parametric maps. Eight physicians were recruited as readers and divided into two groups based on their clinical experience in ultrasound, and all eight readers had no prior access to the data of the 62 cases included in this study:

Junior Group: Four physicians with 1 to 2 years of postgraduate ultrasound experience.

Senior Group: Four physicians with more than 10 years of experience, specializing in urogenital ultrasound.

The reading sessions were conducted as follows:

Session 1 (Conventional Diagnosis): All eight readers, working independently in a quiet environment, reviewed the cases. For each case, they were provided with the grayscale TRUS images and the standard TR-CEUS cineloop. They were blinded to all clinical information, including PSA and patient history, as well as the final pathology. They were asked to provide a binary diagnosis: “benign” or “malignant”.

Washout Period: A minimum interval of two weeks was enforced between the two reading sessions. This washout period was designed to minimize recall bias.

Session 2 (TIC-Assisted Diagnosis): The readers reviewed the same 62 cases, presented in a randomized order. This time, in addition to the grayscale and CEUS images, they were also provided with the corresponding TIC parametric map. They were again asked to provide a final binary diagnosis of “benign” or “malignant”.

### 2.6 Statistical analysis

All statistical analyses were performed using R software (version 4.4.0, R Foundation for Statistical Computing, Vienna, Austria). A p-value of < 0.05 was considered statistically significant for all tests.

Baseline Characteristics & Trend Analysis: Descriptive statistics were used to summarize patient demographics and clinical data. The Cochran-Armitage test for trend was employed to assess whether there was a statistically significant trend between the increasing ordinal levels of the discreteness classification (Type 1 to 4) and the proportion of malignant cases.

Diagnostic Outcome Comparison: A paired chi-square test (McNemar’s test) was used to compare the diagnostic decisions (benign vs. malignant) made with and without TIC assistance for each reader group, to determine if the addition of the parametric map led to a significant change in diagnoses.

Inter-Rater Reliability: To assess diagnostic consistency among the four physicians within each group (junior and senior), the intra-class correlation coefficient (ICC) was calculated for both the conventional and TIC-assisted sessions. The two-way random effects model, assessing for absolute agreement, was used. ICC values were interpreted as follows: < 0.5 (poor), 0.5–0.75 (moderate), 0.75–0.9 (good), and > 0.9 (excellent).

Inter-Group Agreement: Fleiss’ Kappa statistic was used to assess the level of agreement between different diagnostic methods or reader groups (e.g., junior-unassisted vs. senior-unassisted; junior-assisted vs. senior-assisted).

Diagnostic Performance Analysis: The primary analysis of diagnostic accuracy was conducted using Receiver Operating Characteristic (ROC) curves. For each of the four conditions (Junior-Unassisted, Junior-Assisted, Senior-Unassisted, Senior-Assisted), sensitivity, specificity, positive predictive value (PPV), negative predictive value (NPV), and overall accuracy were calculated based on the binary diagnoses. The Area Under the Curve (AUC) was calculated as the main metric of diagnostic performance. The statistical significance of the difference between the AUCs of paired data (e.g., Junior-Unassisted vs. Junior-Assisted) was determined using the DeLong test. Youden’s index (J = Sensitivity + Specificity - 1) was also calculated to identify the optimal balance of sensitivity and specificity.

## 3 Results

### 3.1 Patient and lesion characteristics

As detailed in **Table 1**. The final cohort consisted of 62 male patients with a mean age of 68.4 ± 11.2 years (range: 43 to 90 years). The mean total PSA level was 21.5 ± 28.9 ng/ml (range: 2.99 to 153.94 ng/ml). Histopathological analysis of the 12-core biopsy specimens confirmed prostate cancer in 11 of the 62 patients (17.7%) and benign findings (e.g., BPH, prostatitis, atrophy) in the remaining 51 patients (82.3%).

**Table 1:** Baseline data of patients.

Classification dimensions	Grouping/Grading	Number of cases (n = 61)	Proportion (%)	Average age (years)	Total PSA value range (ng/ml)
Nature of the disease	benign	50	82	67.2	2.99-153.94
	malignant	11	18	71.5	3.22-146
age stratification	Under 60	12	20	54.8	5.11-26.07
	60–69 years	21	34	65.1	4.12-100
	70–79 years	21	34	73.8	3.22-100
	≥ 80 years of age	7	11	83.1	5.11-146
Discreteness grading	Level 1	38	62	66.3	2.99-153.94
	Level 2	12	20	66.8	4.12-21.33
	Level 3	6	10	71.7	12.65-146
	Level 4	5	8	70.6	9.14-146
PSA horizontal	<4	2	3	64.5	2.99-3.22
	45757	20	33	67.6	4.12-9.98
	45950	20	33	68.4	10.06-20.62
	>20	19	31	68.9	21.33-153.94

When classified by the TIC parametric imaging discreteness system, there were 38 (61.3%) Type 1 cases, 12 (19.4%) Type 2 cases, 6 (9.7%) Type 3 cases, and 5 (8.1%) Type 4 cases.

### 3.2 Discreteness classification and correlation with malignancy

**Figure 3:**
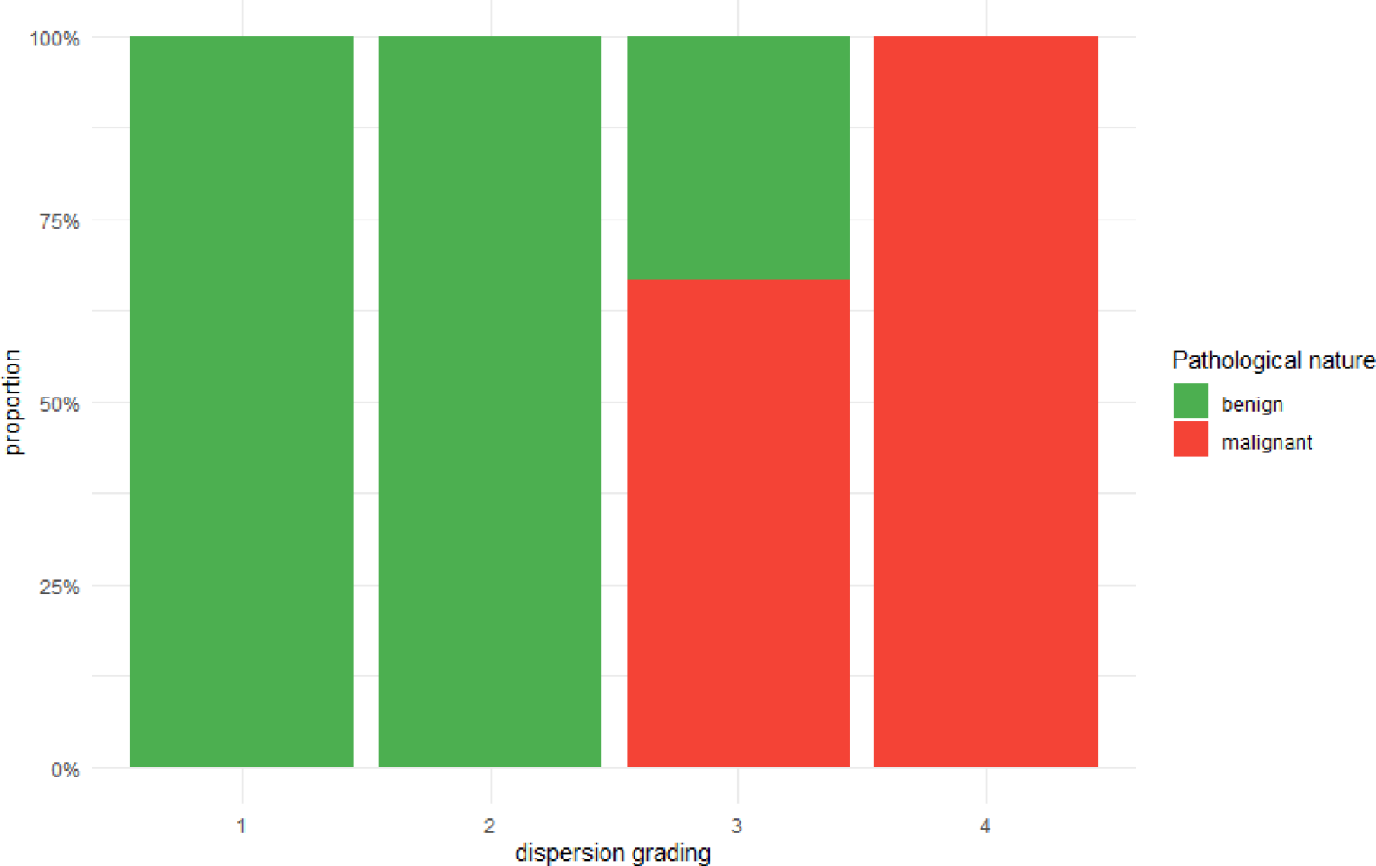
Stacked bar chart of the ratio of benign to malignant with changes in grading.

As detailed in **Figure 3**, A strong positive association was observed between the perfusion discreteness classification and the likelihood of malignancy. Of the 11 cancer cases, 9 (81.8%) were classified as either Type 3 or Type 4. Conversely, of the 51 benign cases, 46 (90.2%) were classified as Type 1 or Type 2. The Cochran-Armitage test for trend confirmed a highly significant trend (Z = 5.5578, p < 0.001), indicating that as the spatial heterogeneity of perfusion (discreteness grade) increases, the probability of a malignant diagnosis rises monotonically.

### 3.3 Improvement in diagnostic consistency

The introduction of TIC parametric maps led to a marked improvement in inter-rater reliability for both physician groups. As detailed in **Table 2**, the intra-group ICC for the four junior physicians, which was already in the “good” range at 0.832 (95% CI: 0.742-0.902) for conventional diagnosis, rose significantly to an “excellent” 0.915 (95% CI: 0.873-0.948) with TIC assistance. This suggests that the objective nature of the parametric map helped standardize their interpretations. For the senior physicians, who already exhibited excellent baseline consistency (ICC = 0.878; 95% CI: 0.802-0.933), the addition of the TIC map further consolidated their agreement, pushing the ICC to a near-perfect 0.941 (95% CI: 0.906-0.966).

**Table 2:** Intra-group consistency analysis.

Groups	ICC price	95% confidence interval	p price
Junior physician	0.832	0.742-0.902	<0.001
Junior physician + TIC assistance	0.915	0.873-0.948	<0.001
Senior physician	0.878	0.802-0.933	<0.001
Senior physician + TIC assistance	0.941	0.906-0.966	<0.001

Inter-group agreement analysis further highlighted the technology’s impact. As detailed in **Table 3**, The baseline agreement between unassisted junior and senior physicians was only “fair” (Kappa = 0.444), underscoring the known discrepancy in diagnostic ability due to experience. Crucially, when both groups used TIC assistance, their diagnostic agreement became “substantial” (Kappa = 0.771), demonstrating that the technology effectively harmonized their diagnostic conclusions and bridged the experience gap.

**Table 3:** Intergroup consistency (Kappa) analysis.

Group 1	Group 2	Kappa	P.value	CI_95
Junior physicians	Junior physicians + TIC assistance	0.125	0.04	[0.084, 0.166]
Junior physicians	Senior physicians	0.444	0.0001	[0.382, 0.506]
Junior physicians + TIC assistance	Senior physicians	0.327	0.0001	[0.269, 0.386]
Junior physicians + TIC assistance	Senior physicians + TIC assistance	0.771	0.0001	[0.719, 0.823]
Senior physicians	Senior physicians + TIC assistance	0.176	0.004	[0.128, 0.223]

### 3.4 Enhancement of diagnostic performance

**Figure 4:**
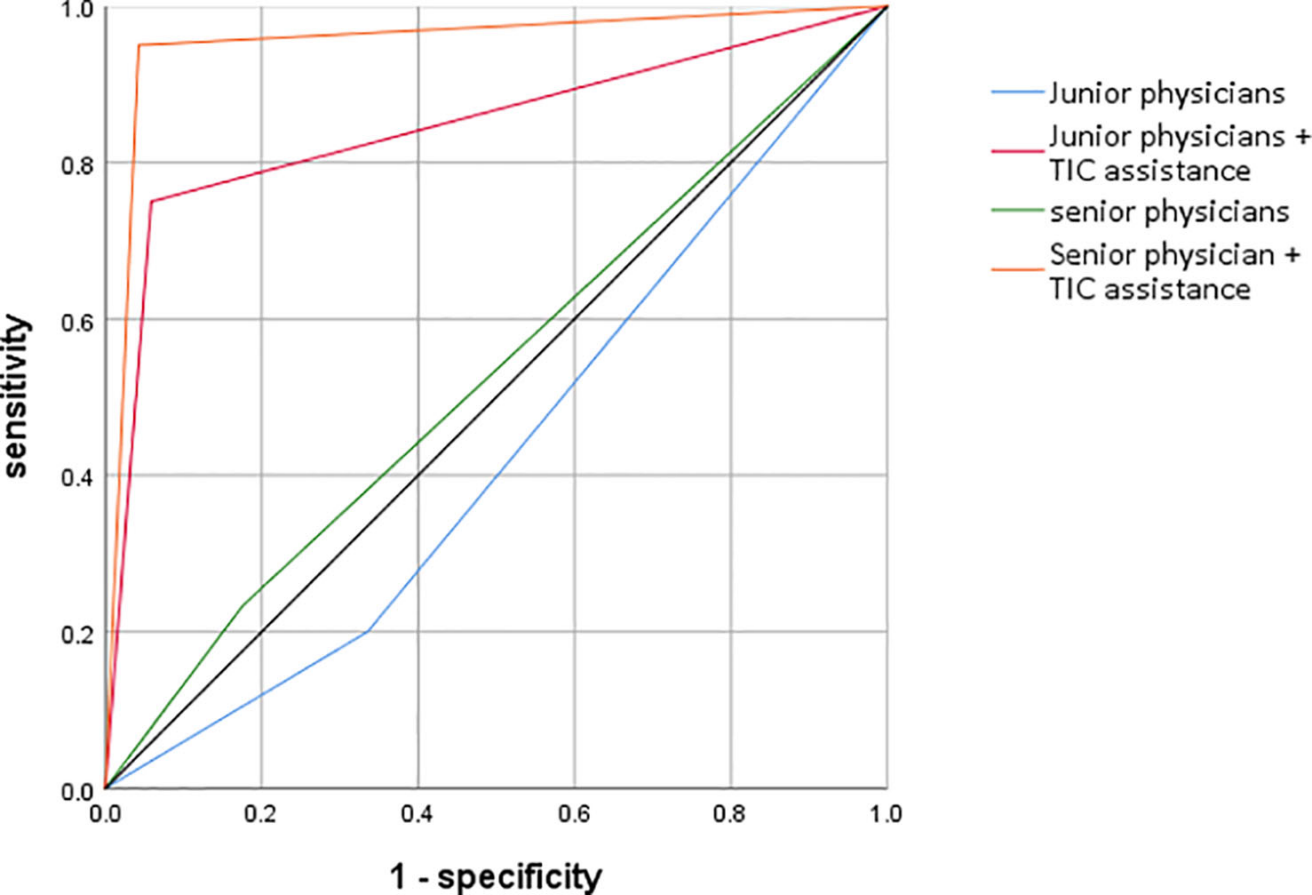
ROC curves for the four diagnostic scenarios.

The most striking finding of the study was the dramatic improvement in diagnostic accuracy for both groups with the use of TIC parametric imaging. The ROC analysis, summarized in **Table 2** and visualized in **Figure 4**, quantifies this effect.

As detailed in **Table 4**. For junior physicians, the unassisted diagnostic performance was poor, with an AUC of only 0.53, barely better than chance. With the aid of the parametric maps, their performance surged to an excellent AUC of 0.89. This improvement was highly statistically significant (p < 0.01),.

**Table 4:** Diagnostic performance metrics and ROC analysis.

Divide into groups	AUC (95%CI)	Sensitivity (95%CI)	Specificity (95%CI)	PPV (95%CI)	NPV (95%CI)
Junior physicians	0.43 (0.36-0.50)	0.20 (0.10-0.35)	0.65(0.57-0.73)	0.16 (0.07-0.27)	0.72 (0.63-0.80)
Junior physicians + TIC assistance	0.85 (0.78-0.91)^a^	0.94 (0.88-0.97)	0.79 (0.64-0.90)	0.92 (0.87-0.96)	0.76 (0.61-0.87)
Senior physicians	0.53 (0.46-0.60)^a,b^	0.29 (0.15-0.48)	0.83 (0.76-0.89)	0.22 (0.11-0.37)	0.77 (0.70-0.83)
Senior physicians + TIC assistance	0.95 (0.92-0.99)^a,b,c^	0.96 (0.93 - 0.99)	0.95 (0.89 - 1.00)	0.98 (0.97 - 1.00)	0.88 (0.80 - 0.96)

^a^indicates the comparison of AUC values between each group and the primary physician’s diagnosis using a paired t-test, with p<0.05; ^b^indicates the comparison of AUC values between each group and the primary physician’s diagnosis assisted by parameter charts using a paired t-test, with p<0.05; ^c^indicates the comparison of AUC values between each group and the senior physician’s diagnosis assisted by parameter charts using a paired t-test, with p<0.05.

For senior physicians, the baseline performance was better but still suboptimal, with an AUC of 0.68. TIC assistance elevated their performance to an outstanding AUC of 0.95, also a highly significant improvement (p < 0.01).

Notably, the TIC-assisted performance of the junior physicians (AUC = 0.89) was not only a vast improvement on their own baseline but also significantly surpassed the unassisted performance of the senior physicians (AUC = 0.68), highlighting the technology’s potential to accelerate the acquisition of diagnostic expertise.

## 4 Discussion

This study demonstrates that a novel TIC parametric imaging technique, which provides a quantitative and visual map of prostate perfusion heterogeneity, serves as a powerful diagnostic adjunct that substantially improves the accuracy and reliability of prostate cancer detection using TR-CEUS. Our key findings—a dramatic increase in diagnostic AUC, a marked improvement in inter-rater reliability, and a significant narrowing of the performance gap between junior and senior physicians—collectively point to the profound clinical potential of this technology.

### 4.1 Overcoming the subjectivity of conventional ultrasound

A long-standing criticism of ultrasound imaging, particularly for diffuse disease processes like PCa, is its dependence on operator experience and subjective interpretation. Conventional TRUS relies on the detection of subtle, often non-specific, hypoechoic lesions (5). TR-CEUS improved upon this by adding functional information, but its interpretation of hypervascularity remains largely qualitative and susceptible to confounders like BPH and prostatitis (6). Previous attempts at quantitative CEUS were hampered by the limitations of manual ROI placement, which is both subjective and incapable of capturing the full spatial complexity of tumor perfusion (12, 13).

Our study directly addresses this challenge. By automating the TIC analysis on a pixel-by-pixel basis across the entire gland, our parametric imaging approach removes the subjectivity of ROI placement and transforms the complex, dynamic data from a 4D cineloop (2D space + time + intensity) into an easily interpretable 2D static image. This objective visualization of perfusion hotspots and their spatial distribution provides a robust, standardized foundation for diagnosis. The significant improvement in the intra-group ICC for both junior (0.832 to 0.915) and senior (0.878 to 0.941) physicians is direct evidence of this standardizing effect. The parametric map provides a common, data-driven visual language that reduces interpretive ambiguity and leads to more consistent conclusions among different observers.

### 4.2 Bridging the experience gap and implications for clinical training

The disparity in diagnostic accuracy between novice and expert physicians is a well-documented phenomenon across many fields of medical imaging (14). Our results starkly illustrate this gap in the context of PCa ultrasound, with unassisted senior physicians (AUC = 0.53) significantly outperforming their junior colleagues (AUC = 0.43). This gap poses a significant challenge for healthcare systems, particularly in regions with a shortage of expert sonographers, potentially leading to delayed diagnoses and suboptimal patient care (15).

The most impactful finding of our study is the ability of TIC parametric imaging to virtually eliminate this experience gap. The assisted performance of junior physicians (AUC = 0.85) was not only a monumental improvement but was statistically comparable to the assisted performance of senior experts (AUC = 0.95). This suggests that the technology acts as a great equalizer, empowering less experienced users with a tool that encapsulates some of the pattern recognition skills of an expert. It provides clear, actionable visual cues that guide the observer to the correct diagnosis. This has profound implications for clinical training and workforce development. TIC parametric imaging system employed in this study is a standalone offline analysis software. Consequently, following the completion of TR-CEUS examinations, sonographers must perform additional operations using this software for subsequent analysis. The workflow involving exporting images from the PACS system to external software for offline processing and interpretation takes at least 30 minutes per case, making it difficult to implement on a large scale within the often heavy workflow of ultrasound examinations. If real-time TIC parametric imaging could be integrated into ultrasound diagnostic systems, enabling instantaneous analysis, it would significantly improve diagnostic efficiency and hold great potential for application in targeted prostate biopsy. However, this study preliminarily validates that the software offers significant value in enhancing the diagnostic accuracy for prostate cancer among junior physicians and shortening their learning curve. A key advantage is that this improvement in efficacy is achieved without requiring additional human resource expenditure. Integrating such tools into residency and fellowship programs could dramatically shorten the learning curve for diagnosing PCa, allowing trainees to achieve expert-level performance more rapidly and confidently.

### 4.3 Biological rationale: imaging the tumor microenvironment

The diagnostic power of our method is rooted in its ability to image the underlying biological process of tumor angiogenesis. Malignant tumors induce the growth of a chaotic, leaky, and heterogeneous vascular network to sustain their growth (11, 16). This pathological neovasculature is functionally distinct from the orderly blood supply of benign tissue. Our chosen parameter, the “mean gradient to peak,” is a sensitive measure of the rate of microbubble influx, directly reflecting the rapid and high-volume flow characteristic of tumor-associated vessels (17).

Furthermore, our four-tier discreteness classification captures the spatial heterogeneity of this aberrant perfusion, which is a key feature of the tumor microenvironment. A growing tumor is not a uniform mass; it is a complex landscape with a hypervascular, proliferating outer rim and often a more hypoxic, sometimes necrotic, core (18). This leads to a spatially disorganized perfusion pattern. Our results strongly support this biological premise: 82% of cancers exhibited a highly discrete (Type 3 or 4) perfusion pattern, whereas 90% of benign cases showed a more focal and organized (Type 1 or 2) pattern. This finding aligns with observations from DCE-MRI and histopathology, which also highlight spatial heterogeneity as a hallmark of malignancy (19, 20), and suggests our classification system is effectively capturing a biologically relevant feature of PCa. Beyond the aforementioned considerations, research utilizing Perturb-DBiT and spatially resolved CITE-seq technologies suggests that the aberrant manifestations observed in prostate cancer TIC parametric imaging may be associated with the following cellular biological and genetic mechanisms:

Spatial Heterogeneity & Genetic Drivers: Perturb-DBiT analysis revealed profound intratumoral spatial heterogeneity within prostate cancer. Genetically, CRISPR screening identified that the loss of genes such as MT1E and S100A4 promotes tumor cell migration. This enhanced migratory capacity potentially contributes to disorganized vascular architecture, which may subsequently impact contrast agent perfusion kinetics observable in TIC imaging (21).

Immune Microenvironment & Vascular Function: High-dimensional protein expression mapping via spatial CITE-seq demonstrated spatial colocalization of CD31+ endothelial cells and immune checkpoint proteins. This spatial association indicates the presence of a local immunosuppressive microenvironment, which could indirectly contribute to alterations in vascular permeability–a factor potentially reflected in TIC parameters (22).

Tph Cells (peripheral helper T cells) & Vascular Modulation: Furthermore, enriched regions of Tph cells, as visualized, coinciding with local immune activation, may alter vascular permeability through cytokine release (23).

### 4.4 Clinical implications and future workflow integration

As detailed in **Table 4**. The high diagnostic accuracy (AUC up to 0.95) and negative predictive value (NPV up to 0.98) of TIC-assisted diagnosis suggest several potential shifts in the clinical workflow for PCa detection. Firstly, it could serve as a highly effective triage tool. For patients with intermediate PSA levels, a negative TIC parametric imaging result (e.g., Type 1 discreteness) could provide greater confidence in deferring an immediate biopsy in favor of continued monitoring, potentially sparing many men from an unnecessary, invasive procedure and its associated risks (24, 25).

Secondly, for patients proceeding to biopsy, the parametric map could be used to guide targeted sampling. Instead of relying on a blind, systematic 12-core approach, the map could be fused with the real-time ultrasound image to direct biopsy needles precisely into the areas of highest perfusion abnormality (the “hot spots”). This approach, analogous to MRI-ultrasound fusion biopsy, could increase the diagnostic yield for clinically significant cancers and provide more accurate Gleason grading by targeting the most aggressive parts of the tumor (26–29). This could be particularly valuable for patients with a negative prior systematic biopsy but persistently elevated PSA, where the cancer may have been missed by blind sampling.

### 4.5 Limitations of the study

Despite the promising results, this study has several limitations that must be acknowledged. First, its retrospective, single-center design may introduce selection and information bias and limits the generalizability of our findings to other patient populations and clinical settings. The retrospective, single-center design of this study inherently limits the external validity of our findings and may introduce biases related to patient selection and operator-dependent assessments. We explicitly acknowledge this as a significant limitation of our work. To address this limitation and significantly improve the credibility and applicability of our results, future research must prioritize prospective multicenter validation. A prospective study would eliminate selection bias by enrolling a consecutive and predefined patient population, thereby providing a more accurate representation of the real-world clinical setting. Furthermore, a multicenter design is crucial for enhancing generalizability. By involving multiple institutions with different patient demographics, varied clinical practices, and diverse imaging equipment, the results would demonstrate robustness across a broader spectrum of scenarios. This would rigorously test whether the diagnostic performance observed in our single-center cohort can be reliably replicated in other, independent populations. Successful validation through such a design would not only confirm the utility of our model but also mark a critical step towards its potential integration into clinical practice, ensuring its findings are both credible and universally applicable.

Second, the sample size is relatively small, particularly the number of cancer cases (n=11), which can lead to wide sensitivity confidence intervals and increases the risk of statistical anomalies. The width of the confidence intervals for sensitivity, particularly, is substantial. For instance, the sensitivity for Senior Physicians ranges from 15% to 48%. This indicates a considerable degree of statistical uncertainty. A model with a point estimate of 29% sensitivity could, in reality, perform as low as 15% or as high as 48% in different populations. This imprecision is a direct consequence of the small event count. A model developed on a dataset with a low number of events is more susceptible to overfitting. This means the model may perform well on the specific data it was trained on but fail to generalize effectively to new, independent datasets. The observed performance may be inflated by learning dataset-specific noise rather than the true underlying biological signals of malignancy. The results, while statistically significant, require validation in larger, multi-center prospective trials.

Third, Current urological guidelines recommend multiparametric magnetic resonance imaging for men with suspected prostate cancer. The consequent increase in MRI demand may impose substantial strain on healthcare systems (30). TIC parametric imaging, which can be integrated into ultrasound-based diagnostic workflows, has the potential to alleviate this burden. On the other hand, the role of TIC parametric imaging is primarily adjunctive; for instance, in equivocal cases such as PI-RADS category 3 lesions, subjective interpretation aided by TIC parametric imaging may upgrade lesions to PI-RADS category 4. In the future, we plan to design head-to-head studies involving multicenter trials to compare the diagnostic performance of “mpMRI” versus “TIC parametric imaging”, with primary endpoints including PCa detection rate or histopathological concordance. A future head-to-head comparison or a fusion analysis combining the strengths of TIC parametric imaging (superior temporal resolution for perfusion) and mpMRI (superior soft-tissue contrast and anatomical detail) would be essential to define the exact role of our technique in the diagnostic algorithm (31).

Fourth, the histopathological reference standard was systematic biopsy, not radical prostatectomy specimens. It is well-known that systematic biopsy is prone to sampling error and can miss or underestimate the extent and grade of tumors (32). The reported sensitivity in this study is likely underestimated. This suggests that the actual ability of the novel method to detect prostate cancer in clinical practice may be superior to the figure reported herein. The specificity and NPV are likely overestimated. Consequently, the ability of the novel method to exclude prostate cancer in real-world applications may be less robust than the results of this study indicate. Therefore, if applied clinically, considerable caution is warranted, and clinicians should not rely solely on a negative result to definitively exclude prostate cancer. Despite these limitations inherent in the reference standard, the primary value of this study resides in its comparative assessment of the novel method against current standard methods, utilizing the same widely accepted (albeit imperfect) reference standard. The conclusion that the novel method demonstrates superior performance relative to existing approaches remains valid and meaningful, even if the absolute values of the performance metrics are potentially biased. Future research should employ more reliable reference standards, such as those incorporating targeted biopsies or, where feasible, radical prostatectomy specimen pathology (33). Additionally, we plan to conduct longitudinal follow-up studies on patients with negative diagnostic results. Monitoring the subsequent prostate cancer detection rate in this cohort will provide indirect assessment of the impact of reference standard sampling error and yield a more accurate estimation of the method’s true NPV. While prostatectomy is the gold standard, it is only available for a subset of patients who undergo surgery.

Finally, while the parametric map itself is quantitative, our four-tier discreteness classification remains a qualitative, subjective assessment. Although it proved effective and showed good inter-rater reliability with training, the development of a fully automated, quantitative algorithm for classifying spatial heterogeneity using machine learning or deep learning would be a critical next step to completely remove observer subjectivity (14, 34).

### 4.6 Future directions

This work opens several exciting avenues for future research. The immediate priority is to conduct a large-scale, multi-center prospective trial to validate our findings. Future studies should incorporate mpMRI to perform head-to-head comparisons and explore the potential of US-MRI fusion imaging guided by TIC parametric maps. The development of a fully automated workflow, incorporating AI-driven prostate segmentation and quantitative heterogeneity analysis, is crucial for clinical translation. Extending this technique to 3D ultrasound would enable whole-gland perfusion analysis, overcoming the limitations of single-plane imaging (35). Lastly, longitudinal studies could explore whether TIC parameters can serve as non-invasive biomarkers for predicting treatment response or monitoring disease progression in patients on active surveillance.

## 5 Conclusion

In conclusion, this study provides compelling evidence that TIC parametric imaging represents a significant technological advancement in the ultrasound diagnosis of prostate cancer. By providing an objective, quantitative, and intuitive visualization of tumor perfusion heterogeneity, this technique dramatically improves diagnostic accuracy, enhances inter-observer reliability, and effectively mitigates the impact of clinical experience. It addresses key limitations of conventional ultrasound and offers a practical, accessible, and powerful tool that has the potential to refine clinical workflows, reduce unnecessary biopsies, and ultimately improve patient outcomes. Further validation through large-scale prospective studies is warranted to facilitate its integration into routine clinical practice.

## Data Availability

The original contributions presented in the study are included in the article/supplementary material. Further inquiries can be directed to the corresponding authors.
